# Microbiota-Immune Interactions in Ulcerative Colitis and Colitis Associated Cancer and Emerging Microbiota-Based Therapies

**DOI:** 10.3390/ijms222111365

**Published:** 2021-10-21

**Authors:** Jelena Popov, Valentina Caputi, Nandini Nandeesha, David Avelar Rodriguez, Nikhil Pai

**Affiliations:** 1 Division of Pediatric Gastroenterology and Nutrition, Department of Pediatrics, McMaster University, Hamilton, ON L8S 4L8, Canada; popovj2@mcmaster.ca; 2 College of Medicine and Health, University College Cork, T12 XF62 Cork, Ireland; 3 Department of Poultry Science, University of Arkansas, Fayetteville, AR 72701, USA; vcaputi@uark.edu; 4 School of Medicine, Royal College of Surgeons in Ireland, D02 YN77 Dublin, Ireland; nandininandeesha@rsci.com; 5 Department of Pediatrics, University of Toronto, Toronto, ON M5S 1A1, Canada; david.avelarrodriguez@sickkids.ca; 6 Farncombe Family Digestive Health Research Institute, McMaster University, Hamilton, ON L8S 4L8, Canada

**Keywords:** ulcerative colitis, inflammatory bowel diseases, pediatrics, FMT, probiotics, synbiotics, antibiotics, prebiotics, fecal microbiota transplant, colitis-associated cancer, colorectal cancer, CAC, CRC, dysbiosis

## Abstract

Ulcerative colitis (UC) is a chronic autoimmune disorder affecting the colonic mucosa. UC is a subtype of inflammatory bowel disease along with Crohn’s disease and presents with varying extraintestinal manifestations. No single etiology for UC has been found, but a combination of genetic and environmental factors is suspected. Research has focused on the role of intestinal dysbiosis in the pathogenesis of UC, including the effects of dysbiosis on the integrity of the colonic mucosal barrier, priming and regulation of the host immune system, chronic inflammation, and progression to tumorigenesis. Characterization of key microbial taxa and their implications in the pathogenesis of UC and colitis-associated cancer (CAC) may present opportunities for modulating intestinal inflammation through microbial-targeted therapies. In this review, we discuss the microbiota-immune crosstalk in UC and CAC, as well as the evolution of microbiota-based therapies.

## 1. Background

Inflammatory bowel disease (IBD) is a chronic autoimmune condition affecting the gastrointestinal (GI) tract. It comprises Crohn’s disease (CD) and ulcerative colitis (UC), and generally presents as a progressive inflammatory condition. UC is characterized by inflammation of colonic mucosa and submucosa starting at the rectum and extending through the colon. Typical symptoms of UC flares include abdominal pain, hematochezia, tenesmus, and loose stools. Extraintestinal manifestations may also present, including ocular pathologies, arthropathies, liver disease such as primary sclerosing cholangitis, and dermatological manifestations [1].

Various genetic and environmental factors have been implicated in UC susceptibility [2]. To date, over 200 single nucleotide polymorphisms (SNPs) have been associated with the risk of developing UC [3]. Epidemiological studies have shown a higher incidence of UC among populations adopting Western diets rich in refined sugars, dairy, protein, and animal fat, and low in dietary fiber including wholegrains, fruits, and vegetables [4]. The role of environmental influences aligns with the hygiene hypothesis, which states that limited exposure to microorganisms during infancy and childhood may impair appropriate priming and development of the immune system, thus promoting autoimmunity [5]. Exposure to antibiotics during gestation and childhood, psychological stress, and family history also affect the risk of developing UC [6]. These factors profoundly alter the intestinal microbiome but may also provide opportunities for new treatment options. 

The extent and duration of UC disease activity is associated with an increased risk of neoplasia [7]. The risk of developing colitis-associated cancer (CAC) begins increasing 8 to 10 years after UC diagnosis [8]. Previous studies have estimated a risk of 2% by 10 years, 8% by 20 years, and 18% by 30 years [7]. Other studies have shown that while sporadic colorectal cancer (CRC) affects 1–2% of the general population, over 13% of patients with UC will develop CAC [9]. This corresponds to a 4 to 10-fold increased incidence as compared with sporadic CRC [8]. Sex differences have also been reported, with higher CAC prevalence and mortality rates observed among male patients [10]. The relationship between UC and CAC has influenced the development of clinical practice guidelines, with increased endoscopic surveillance recommended among UC patients starting 8 years after initial UC diagnosis. These recommendations have led to successful reductions in CAC morbidity and mortality [11].

The mechanisms underpinning UC pathogenesis remain unclear, but the dominant hypothesis suggests that environmental factors, including alterations in intestinal microbiota, contribute to an exaggerated immune response and chronic inflammation in genetically susceptible individuals [12]. Conventional treatments for UC have largely relied on dampening the immune response to induce disease remission and promote mucosal healing [13]. Pharmacotherapies such as corticosteroids and disease modifying anti-rheumatic drugs remain the dominant treatment paradigm; however, these medications have significant side effect profiles and may induce immune tolerance with long term use. These medications are also associated with significant healthcare costs, particularly newer biological therapies which require ongoing dosing. Emerging therapies have focused on the potential benefits of microbiota-targeted alternatives, including prebiotics, probiotics, synbiotics, antibiotics, and fecal microbiota transplantation (FMT). This review will discuss key changes in intestinal microbiota associated with UC pathogenesis and immune dysfunction, as well as the role of microbiota-based therapies in affecting intestinal inflammation and progression to neoplasia.

## 2. Microbiome-Immune Interactions in UC 

### 2.1. Immune System Perturbations in UC 

**Figure 1:**
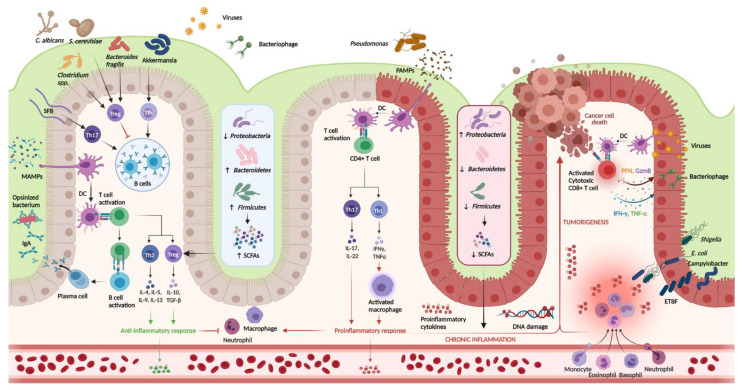
Host-immune interactions in ulcerative colitis. Intestinal microbiota interact with the immune system through various pathways. In the healthy colon, DCs sample MAMPs and present antigens on major histocompatibility complex class II to naive CD4+ T cells. Naive CD4+ T cells become activated and differentiate into various T cell subtypes depending on the presence of specific cytokines within the local microenvironment. Anti-inflammatory Th subtypes comprise Th2 and Treg cells. CD4+ T cells also activate plasma cells which secrete immunoglobulin A (IgA) which is essential for microbial opsonization. Proinflammatory Th subtypes consist of Th1 cells and Th17 cells, which are upregulated in the diseased colon via interactions between DCs and PAMPs. Chronic inflammation contributes to DNA damage and tumorigenesis. Invading viruses stimulate CD8+ cytotoxic T cell activation via antigen-MHC I interactions. However, CD8+ T cells can also assist in cancer cell death. Disruptions in the mucosal barrier provides avenues for microbial translocation, including ETBF, which has been implicated in colitis-associated cancer. Finally, the production of SCFA is increased in the healthy colon (mediated by increased density of Firmicutes and Bacteroidetes phyla), while increased density of the Proteobacterium phylum is associated with lower concentrations of SCFA and colonic inflammation. DC, dendritic cell; DNA, deoxyribonucleic acid; ETBF, enterotoxigenic *Bacteroides fragilis*; IFN-γ, interferon-gamma; IgA, immunoglobulin A; MAMPs, microbe-associated molecular patterns; PAMPs, pathogen-associated molecular patterns; SCFAs, short chain fatty acids; SFB, segmented filamentous bacteria; Th, T helper; Treg, T regulatory; TNF-α, tumor necrosis factor-alpha. Created in Biorender.com (accessed: 1 August 2021) [16].

Perturbations in intestinal microbiota and immune dysregulation are key features of UC pathogenesis (Figure 1). Colonization is largely believed to commence during parturition, although limited evidence suggests that some microbial cells might be present in utero during the prenatal period [14]. The largest contributors to intestinal microbiota composition constitute mode of childbirth and feeding during infancy. Subsequent expansion and diversification of the intestinal microbiome continues throughout childhood and adolescence until a relatively stable composition is achieved in adulthood [15].

Early life may be considered a common denominator between intestinal microbiota development and susceptibility to UC, as perturbations in early microbial colonization such as caesarean section delivery, dietary changes, exposure to antibiotics, systemic stressors, and infection constitute the same environmental factors associated with the risk of developing UC [17,18,19].

The microbiome plays an important role in maintaining intestinal homeostasis by training the innate and adaptive immune systems to tolerate commensal microbes, while offering protection against harmful pathogens [20,21,22]. Tolerance towards commensal microorganisms is mediated via: (1) reducing contact between luminal microbes and the intestinal mucosa through physical barriers [23], and (2) development of immune hyporesponsiveness [24].

The intestinal mucosal barrier serves as the first line of defense against bacterial translocation into systemic circulation and is composed of physical and immunological elements working together to maintain intestinal health. Alterations in the physiological composition of gut microbes in early life disrupt tolerance to commensals, permit translocation of pathogens, and result in dysregulation of host immune function through various signaling cascades [25]. Microbial dysbiosis, intestinal barrier defects, and alterations in mucin secretion may occur even in the absence of active inflammation, including outside of the colon in UC. This suggests that disruptions to normal intestinal physiology are primary contributors to UC pathogenesis and likely predate inflammation [26].

#### 2.1.1. Physical Barrier

A mucus blanket composed of heavily glycosylated mucins serves as the first physical element of the intestinal mucosal barrier. Mucins may be membrane tethered, secretory, or non-gel forming. Their production and secretion are principally mediated by goblet cells and may be influenced by nonspecific factors such as immune system interactions with microbiota and dietary factors, and specific modulators including epigenetics and transcriptional factors [27]. Among the various pathogen recognition receptor (PRR) ligands, toll-like receptor (TLR) ligands serve as particularly powerful stimuli for goblet cell production of mucins [28]. Intestinal microorganisms synthesize a variety of conserved structural components which act as ligands for PRRs termed microbe-associated molecular patterns (MAMPs), which are expressed by commensals and enteropathogens. In the context of pathobionts, MAMPs are typically referred to as pathogen-associated molecular patterns (PAMPs) [29]. Gram-negative bacteria such as *Escherichia coli* and *Pseudomonas aeruginosa* produce PAMPs, lipopolysaccharide (LPS) and flagellin, which bind TLR4 and TLR5, respectively, to alter mucin production and activate inflammatory pathways such as the nuclear factor-κB (NF-κB) cascade. While goblet cells are found throughout the GI tract, they are most concentrated in the colon and rectum where they form a thick mucin bilayer [24,30]. Notably, this increasing density gradient of goblet cells correlates with the density and diversity of gut microbes from proximal to distal aspects of the GI tract [31]. 

The mucous bilayer in the colon consists of a loosely arranged outer layer (ranging in thickness from 100 to 400 µm in the small bowel, to ~700 µm in the colon) which interacts with microbes, and a dense, impenetrable inner layer (ranging in thickness from 15 to 30 µm in the small bowel, to ∼100 µm in the colon) rich in antimicrobial peptides [27,32]. This mucin meshwork allows for selective diffusion of nutrients and oxygen while limiting microbial contact with the underlying epithelium. Glycosylation of mucins is essential for maintaining intestinal homeostasis and involves either *O*-glycosylation or *N*-glycosylation. *O*-glycans act as important food sources for intestinal microbiota, while *N*-glycans maintain the mucosal barrier. Together, these carbohydrate moieties influence the composition of the intestinal microbiota and protect against intestinal inflammation and disease susceptibility [27]. For example, increased glycosylation of *N*-glycans via overexpression of the enzyme β-1,4-galactosyltransferase I (βGalT1) results in a higher Firmicutes to Bacteroidetes ratio, protection against tumor necrosis factor-α (TNF-α) induced inflammation, and decreased susceptibility to dextran sulfate sodium (DSS)-induced colitis [33]. In contrast, reductions in goblet cell densities [34], alterations in mucin production, and discontinuity of the mucous blanket layer have been implicated in UC pathophysiology. Specifically, reduced expression of MUC9 and MUC20, and increases in MUCH16 have been reported across UC patients irrespective of disease activity, while increases in MUC1 and decreases in MUC2 expression appear to be limited to regions of ulceration [1,35,36]. Decreases in mucin glycosylation and sulphation and increases in sialylation impair barrier function and are well described features of UC [37].

Below the mucin layer, the GI tract is lined by a monolayer of intestinal epithelial cells (IECs) connected via junctional complexes, forming villi and crypts. The IECs form the largest physical barrier of the GI tract and are the strongest determinants of protection against the external environment. They physically separate the products of the intestinal lumen from the underlying lamina propria, thereby maintaining intestinal homeostasis. The junctional complexes which connect the IECs are vital in regulating selective transportation of water and nutrients and preventing penetration of the intestinal mucosa by commensals and enteropathogens [38]. These protein complexes are composed of tight junctions, adherens junctions, and desmosomes. The IECs comprise five distinct cell types, including enterocytes, enteroendocrine cells, tuft cells, Paneth cells, and microfold (M) cells [28], which are regenerated by pluripotent stem cells residing within the intestinal crypts [39]. While IECs exhibit primarily protective functions, defects in this barrier layer have been associated with increased susceptibility to gastrointestinal disease. For example, alterations in deoxyribonucleic acid (DNA) methylation and transcriptome patterns have been implicated in UC pathogenesis. Several of the affected pathways include innate immune system function including cytokine signaling and complement activation, as well as extracellular matrix composition including collagen, laminin, and fibril synthesis and degradation [40]. Many of these epigenetic alterations in methylation patterns appear to be independent of microscopic mucosal inflammation and remain stable over time in UC patients. IECs harvested from inflamed mucosa of UC patients exhibit alterations in molecular signaling cascades, including enhanced Notch signaling and TNF-α induced NF-κB signaling [41]. Furthermore, IECs harvested from patients with active UC exhibit higher apoptotic indices which contributes to impaired barrier function and permits translocation of commensal and enteropathogenic microorganisms, resulting in higher levels of proinflammatory cytokines including TNF-α [42]. Increases in TNF-α result in impairment of the mucosal barrier by inducing caspase-dependent apoptosis and caspase-independent necroptosis of multiple IECs [43]. This, in part, explains the therapeutic success of antibodies targeting TNF-α in select patients. However, a subgroup of patients demonstrates little to no response despite adequate dosing and duration of anti-TNF-α treatment, suggesting that intestinal inflammation independent of TNF-α signaling may be involved in certain subgroups of UC patients [44].

#### 2.1.2. Immunoglobulin A

Within the mucus layer reside additional components of the host defense system including antibacterial peptides and secretory immunoglobulin-A (IgA). The gut mucosa harbors the largest concentration of IgA in the human body, which can be produced in a T-cell dependent or T-cell independent manner [45]. Plasma cells within the lamina propria produce dimeric IgA which is shuttled from the basolateral membrane to the apical surface of IECs via the polymeric immunoglobulin receptor (pIgR) [46]. At the apical surface of IECs, the pIgR-Ig complex is cleaved to produce secretory IgA. Once secreted, IgA can mediate its physiological functions including neutralizing bacterial toxins, inhibiting epithelial translocation of PAMPs such as *Shigella* LPS, coating microorganisms to reduce their immunogenicity, and facilitating the uptake of organisms (such as non-invasive *Salmonella*) to stimulate stronger adaptive immune responses [47]. Secretory IgA is essential for protecting against microbial invasion, influencing the composition of intestinal microbiota and protecting against intestinal inflammation [46].

The expression of IgA and pIgR can be altered by the intestinal microbiota. Upregulation can be achieved via activation of the NF-κB signaling cascade through commensal bacteria including *Bacteroides thetaiotaomicron* and certain strains belonging to the *Enterobacteriaceae* family [48,49]. This upregulation is presumably mediated via direct interactions between commensal MAMPs and TLRs, which stimulate myeloid differentiation factor 88 (MyD88) signaling and increase transcription of pIgR [50]. While proinflammatory cytokines such as interferon (IFN)-γ, TNF-α, interleukin (IL)-1, and IL-4 induce pIgR transcription, paradoxically, intestinal inflammation associated with UC causes downregulation of pIgR expression by IECs [51]. In addition to downregulating pIgR expression, UC is associated with lower concentrations of secretory IgA in the intestinal lumen, higher concentrations of IgA in the serum, decreased transcytosis of dimeric IgA across IECs, and accumulation of IgA within the lamina propria [51].

Crosslinking of IgA with its cognate transmembrane receptor on neutrophils, i.e., FcαRI, stimulates neutrophil recruitment to inflamed tissues and stimulates the release of leukotriene B4 (LTB4), a potent neutrophil chemoattractant [52]. In this manner, a sustained inflammatory loop can be maintained leading to excessive tissue damage. In addition to increased IgA–FcαRI interactions, UC disease activity is also associated with increased neutrophil uptake of IgA-opsonized bacteria within the intestinal mucosa [52]. This contributes to lower concentrations of IgA within the intestinal lumen, diminished immune protection against enteropathogenic invasion, increasing patient susceptibility to inflammation mediated by microbes, and worsened disease activity. Downregulation of pIgR and somatic mutations in IL-17 signaling have been reported in sporadic CRC, which may be driven by particular members of colonic microbiota [53,54]. The influence of microbiota on tumorigenesis is discussed further below.

#### 2.1.3. Innate and Adaptive Immunity

Within the lamina propria are additional bacterial defenses belonging to innate and adaptive immunity. Innate immunity comprises antibacterial peptides, lysozymes, macrophages, and dendritic cells, while adaptive immunity includes T and B cells, which are concentrated within highly organized lymphoid follicles known as Peyer’s patches [55]. Dendritic cells extend their cytoplasmic projections into the intestinal lumen, where they sample intestinal contents and present antigens to T cells within the Peyer’s patches [56]. These dendritic cells are a heterogenous group of antigen-presenting cells with unique biological function that are primarily focused on maintaining a balance between proinflammatory and tolerogenic responses [57].

Genome-wide association studies have identified over 200 loci specifically associated with increased risk of developing UC [3]. Many of these genes have been implicated in innate and adaptive immune system function and impaired autophagy, including specific defects in extracellular matrix protein 1 (ECM1), IL-10, and IL-23R [58].This impaired clearance of microbes causes persistent stimulation of the innate immunity system, prolonged stimulation of the adaptive immune system and chronic inflammation [59]. Inflamed mucosa exhibits upregulation of TLR2 and TLR4 in dendritic cells, which contributes to increased expression of proinflammatory cytokine IL-12 and alterations in microbial interactions [60]. Activated dendritic cells initiate and perpetuate inflammation alone or in combination with adaptive immune cells [57]. Upregulation of IL-13 receptor subunit α-2 (IL-13Rα2) has also been described in intestinal epithelial cells during active UC, which appears to impair goblet cell function, inhibit mucosal regeneration, and alter IL-13 signaling [61]. While low levels of IL-13 are secreted by natural killer cells and macrophages in non-inflamed colonic mucosa, increased release of IL-13 by mononuclear cells in active UC has been implicated in epithelial cell apoptosis and impairment of tight junctions, subsequently producing conduits for microbial translocation and perpetuation of intestinal inflammation [62].

Commensal microorganisms also produce an abundance of PRR ligands which shape homeostatic immune function. IL-17-producing CD4+ Th17 cells are concentrated within the lamina propria and their immunomodulatory role is highly influenced by commensal bacteria, such as segmented filamentous bacteria (SFB) and *Bifidobacterium adolescentis* [63]. *Bacteroides fragilis*, which is another commensal bacterium, synthesizes a capsular polysaccharide A (PSA) with potent immunomodulatory roles. This PSA contributes to the activation of the phosphoinositide 3-kinase (PI3K) pathway and downstream cAMP response element-binding protein (CREB)-dependent transcription of anti-inflammatory genes [64]. This supports the priming of CD4+ regulatory T (Treg) cells, production of anti-inflammatory IL-10, immune system maturation, and maintenance of Th1/Th2 balance [65]. These host–microbial interactions underscore how early life exposure to microorganisms is critical for shaping host immune interactions, establishing immunoregulatory networks, and influencing susceptibility to inflammatory diseases in later life.

### 2.2. Intestinal Microbiota Composition in Ulcerative Colitis

The vast majority of commensal microbiota are found within the GI tract [66]. Alterations in the structure or function of one or multiple classes of microbes, a condition called microbial dysbiosis, may significantly impact host health and has been implicated in various acute and chronic intestinal disorders such as UC [67].

Gut microbes are uniquely distributed across the GI tract with abundance and composition reflecting varying physiologic conditions. Factors such as pH, luminal transit time, nutritional substrates, and mucus layer composition impact microbial colonization and proliferation [20]. Intestinal microbiota are also fundamental for nutrient extraction, complementing host metabolism, supporting host nutrition and growth, and promoting intestinal cell proliferation by providing a unique enzymatic pool to digest macromolecules derived from dietary sources. Among these, the generation of key metabolites such as short-chain fatty acids (SCFAs), vitamins (i.e., vitamin K, B12), folate and bile acids rely on bacterial metabolism [20]. Several gut microbes possess enzymatic machinery to synthesize or modify host neurotransmitters and hormones [68].

The intestinal epithelium represents a key host-microbe interface in UC. Several studies have demonstrated that the inflammatory processes triggering UC are caused by direct contact of dysbiotic microbes with the intestinal mucosa [69]. To better understand the role of the intestinal microbiota in driving inflammatory processes in UC, the bacterial taxonomic profiles and fungi of stool samples and mucosal biopsies of UC patients have been sequenced [70]. While this phylogenetic analysis presents some limitations due to the intra- and interindividual variability of intestinal microbial communities, multiple studies have reported consistent alterations in the intestinal microbiota of UC patients as compared with healthy controls (Table 1). For example, the microbiome in UC is characterized by reduced bacterial α-diversity, reflecting species richness and evenness, and β-diversity (variability) in community composition between UC and healthy subjects [71,72]. UC is associated with a decrease in the number of bacterial taxa from the Firmicutes and Bacteroidetes phyla and a significant increase in bacterial communities from the Proteobacteria phylum [71,72,73,74,75]. These changes are collectively described as a state of bacterial dysbiosis. This dysbiosis could explain the presence of inflammation in the colon of UC patients, as the increased abundance of Gram-negative taxa such as *Escherichia-Shigella*, *Fusobacterium*, *Actinobacillus*, *Streptococcus,* and *Campylobacter* shift the host-microbe equilibrium towards a proinflammatory phenotype, supported by evidence of altered expression of several TLRs in subjects with UC [76,77,78]. TLR4 recognizes molecular profiles derived from Gram-negative bacteria (i.e., lipopolysaccharide), thus, playing a key role in limiting their invasion when the intestinal barrier is disrupted during inflammation [75]. Conversely, the depletion of members from the *Clostridiaceae* family (phylum Firmicutes), such as *Faecalibacterium prausnitzii* and other species from the genera *Clostridium*, *Ruminococcus*, *Eubacterium*, *Roseburia*, and *Akkermansia* significantly lower production of butyrate, propionate, and acetate, and thus impair epithelial barrier function by reducing colonocyte proliferation and affecting Treg cells’ maturation through abnormal production of proinflammatory markers [20,71,72,79,80,81]. 

*Enterobacteriaceae* (phylum Proteobacteria) uptake carbohydrates from the mucus layer, expanding their colonization and abundance while impairing mucosal integrity [20]. Increased *Enterobacteriaceae* and a lower concentration of *Bacteroides* observed in colonic or rectal UC-biopsies have been associated with inflammation severity and outcomes of relapse and remission [86]. *Bacteroides* suppress inflammation mediated by Th1 and Th2 immune cell activity, whereas the abnormal interaction between *Enterobacteriaceae* or their metabolites with the colonic epithelial cells stimulates the production of proinflammatory cytokines and induces the immune response [86]. Pathogen-induced acute enteritis has also been associated with risk of developing UC. For instance, it has been shown that specific strains of *Campylobacter jejuni* can cause the translocation of non-pathogenic commensal microbes across the intestinal epithelium by disrupting the integrity of the tight junctions. The passage of commensals through the intestinal barrier can increase the number of interactions between such microbes and host immune receptors, including TLRs, resulting in chronic inflammation [92].

In addition to bacterial dysbiosis, UC has also been associated with its own microbiome changes, highlighting the complexity of untangling microbial crosstalk in the pathogenesis of the disease [70,89]. This also extends to the intestinal (fungal) mycobiome. UC patients during active disease show an increase in the Basidiomycota/Ascomycota ratio as compared with those in remission and healthy controls. Sokol et al. found changes in the abundance of *Saccharomyces cerevisiae* and *Candida albicans* in stool samples from UC subjects. The authors also described the ability of *Saccharomyces cerevisiae* to produce anti-inflammatory IL-10, suggesting a role for this yeast in the pathogenesis of gut inflammation. Interestingly, this study reported the presence of strong correlations between fungi and bacteria only in UC and not in CD subjects, highlighting how such interkingdom interactions can enhance and contribute to the inflammatory phenotype of UC [70]. Subsequently, Qiu et al. showed an increase of *Aspergillus* in colonic mucosa specimens from UC subjects. Although this study did not find the same changes in the fungal population observed by Sokol et al., it reported positive correlations between *Wickerhamomyces*, *Penicillium*, and proinflammatory markers. Our knowledge of the host-fungi relationship in inflammation continues to develop [88].

A metagenomic analysis may provide more reliable information regarding the functional role of the intestinal microbiota in UC than taxonomic profiling, as the functional potential of the microbial genome is more stable and conserved [78,83]. Shotgun metagenomics have identified more than 20,000 gene families and up to 15 metabolic pathways altered in UC subjects (Table 1) [78]. UC is associated with a significant increase in protease and peptidase activity, suggesting a bacterial proteolytic signature involved in driving inflammation. Hence, elastase activity negatively correlates with beneficial bacteria such as *Adlercreutzia* and *Akkermansia*, but positively correlates with *Bacteroides vulgatus*, a bacterial species known for its proteolytic functional profile. These findings suggest that fecal proteolytic activity might be predictive of disease outcomes in UC [78].

Recent advances that have allowed sequencing of whole DNA of intestinal microorganisms have also facilitated the exploration of the virus kingdom within the human microbiome. In line with previous findings, UC is associated with compositional and functional changes of the mucosal virobiota [90,93]. In healthy conditions, the intestinal mucosal layer has a relatively low viral load, composed of a diverse viral population that is relatively stable over time. In contrast, UC-colonic biopsies show an expansion of viral abundance and reduced α-diversity of the viral population, which is mainly enriched by Gram-negative bacteriophages, mostly from the Caudovirales order [90]. The parallel viral and bacterial dysbiosis in UC suggest the presence of functional inter-kingdom crosstalk in sustaining inflammatory processes. The enrichment of Gram-negative bacterial taxa observed in UC could potentially stimulate the expansion of bacteriophages against such bacteria, resulting in bacteriolysis and subsequent release of PAMPs that could trigger inflammatory responses [20,90,91].

Despite recent advances in sequencing technologies, further studies are needed to elucidate the causal role of the intestinal microbiota in modulating the inflammatory processes in UC. This may occur by integrating microbiome sciences with metabolomics and epigenetics [83,94]. Understanding the contribution of each microbial kingdom to host–microbe interactions could significantly improve the management of UC and support opportunities for personalized medicine [95].

## 3. Therapeutic Implications of Modifying the Intestinal Microbiome in the Treatment of Ulcerative Colitis 

### 3.1. Prebiotics 

**Figure 2:**
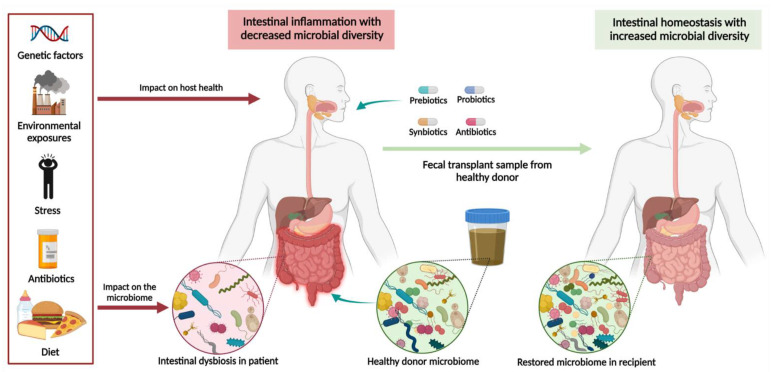
Microbiota-based therapeutic approaches in ulcerative colitis. Various factors have been implicated in contributing to intestinal dysbiosis including a high-fat or low-fiber diet, exposure to antibiotics in early life, psychological stress, environmental pollutants, and genetic factors. Profound disruptions to intestinal microbiota increase an individual’s susceptibility to developing autoimmune disease, including UC. Increasing research is focusing on the role of prebiotics, probiotics, synbiotics, antibiotics, and fecal microbiota transplantation in restoring intestinal homeostasis. While antibiotic exposure in early life increases the risk of developing UC, certain classes of antibiotics may also be used as a therapy once disease is established. Created in Biorender.com (accessed: 1 August 2021) [16].

Prebiotics are defined as nonviable, nondigestive food ingredients which can increase the composition, viability, and growth of beneficial microorganisms (Figure 2) [96]. Prebiotics most commonly comprise inulin or oligosaccharides such as fructans, fructooligosaccharides, galactooligosaccharides, and trans-galactooligosaccharides. Fermentation of prebiotics by intestinal microorganisms generates SCFAs such as butyrate, acetate, and propionate, which are primary nutritional substrates for colonocytes [97]. These SCFAs also have multiple beneficial effects on immune system function and intestinal homeostasis and can act as ligands to G-protein coupled receptors which regulate diverse intestinal functions [98]. Prebiotics may also exert additional metabolic effects on metal ion absorption and fatty acid metabolism and enhance host immunity through upregulation of secretory IgA and cytokine production.

Several studies have described the role of prebiotic preparations in the management of UC. Germinated barley foodstuff high in glutamine and hemicellulose is metabolized by *Eubacterium* and *Bifidobacterium* into butyrate [99]. Butyrate, along with other SCFA, has been shown to play an important role in promoting remission in active UC and is found in significantly lower concentrations in patients with active IBD [100]. Germinated barley foodstuff has also been implicated in inhibiting inflammation mediated by cytokines IL-6, IL-8, and TNF-α, reducing C-reactive protein, and promoting mucosal regeneration [99,101]. Intake of oligofructose-enriched inulin has also been associated with reductions in fecal calprotectin [102].

### 3.2. Probiotics 

Probiotics comprise live microorganisms that may confer important health benefits to the host when consumed [103]. Commensal bacteria found throughout the GI tract help protect against disease-causing pathogen invasion, synthesize and secrete vitamin B12 and vitamin K, promote immune system priming and maturation, and support the production of SCFA [104]. Probiotics also demonstrate antimicrobial properties, mediated through the reduction of intestinal pH via SCFA production, and downregulation of NF-κB signaling in macrophages by butyrate in UC [100,105]. Collectively, these actions reduce the expression of downstream inflammatory mediators such as TNF-α, IL-6, and IL-12. Probiotics such as *Lactobacillus rhamnosus* GG also appear to promote epithelial cell survival and growth by stimulating protein kinase B (PKB) and inhibiting TNF-α mediated apoptosis [106].

While several RCTs have explored the role of probiotics on inducing and maintaining remission in UC, these findings are limited by small sample size and study design. A recent meta-analysis involving 11 RCTs showed that while probiotics pose no serious adverse events for patients as compared with a placebo, there is low certainty of evidence to support their role in maintaining disease remission for UC [107].

VSL#3 and *E. coli* Nissle 1917 have shown the greatest promise for treating UC. VSL#3 is a probiotic cocktail which confers anti-inflammatory benefits via upregulation of IL-10, which inhibits IL-12, IFN-γ, and TNF-α [108]. VSL#3 has also been shown to promote intestinal tight junction integrity and repair of zonula occludens 1 (ZO-1) and occludin post injury via upregulation of the tyrosine-protein phosphatase non-receptor type 2 (PTPN2) gene previously shown to confer protection against IBD [109]. *E. coli* Nissle 1917 reduces colonic inflammation mediated by TNF-α, IL-6, IL-1β, and IL-17, and strengthens the tight junctions which connect IECs [110,111]. Recent studies have suggested that this probiotic may have the potential for toxicity, even at low doses. *E. coli* Nissle 1917 carries the *pks* pathogenicity island in its genome, which codes for a genotoxin suspected to play a role in colorectal cancer development (colibactin). While human data showing detectable levels of colibactin in recipients of *E. coli* Nissle 1917 are lacking, the possibility of long-term adverse effects should be considered [110].

### 3.3. Synbiotics 

Synbiotics are defined as products containing both probiotics and prebiotics, carefully selected to enhance the viability and growth of beneficial microorganisms within the host. The synergistic actions of ingesting both products simultaneously may carry greater therapeutic potential than either product alone [13]. Synbiotic combinations most commonly include *Bifidobacterium longum* and *Lactobacillus rhamnosus* with inulin, fructooligosaccharide, and psyllium [104]. Synbiotics appear to exhibit superior abilities in promoting commensal survival and increased production of SCFAs [111].

A small number of studies have investigated the therapeutic efficacy of synbiotics in the treatment of UC [13]. Among the published data, synbiotics have been shown to decrease CRP [112,113], reduce levels of TNF-α, IL-1β, and IL-8 [114,115], and decrease symptom severity, frequency of short-term disease relapse, and increase duration of remission [115]. While these findings are encouraging, caution should be taken in the interpretation of these results due to small sample size, inconsistent dosing across various studies, and limited availability of placebo-controlled trials. Further randomized controlled trials (RCT) with larger sample sizes are required to assess the impact of synbiotics more effectively in UC treatment.

### 3.4. Antibiotics

Antibiotics have been included in UC therapy as adjuvants, both in the presence of active bacterial infection, and for their ability to suppress the abnormal proliferation of pathogens and stabilize the luminal and mucosal microbial load in favor of the growth of beneficial bacteria [116]. The most commonly prescribed antibiotic agents include inhibitors of cell wall biosynthesis (amoxicillin, vancomycin, and fosfomycin), and inhibitors of nucleic acids (metronidazole and rifaximin) or protein synthesis (tobramycin and vancomycin) [117,118]. Recent studies have shown that combinations of antibiotics, orally administered from 7 days to 3 months, are more effective than single agents alone for improving clinical outcomes in patients with mild to moderate UC [117,119,120,121]. When used in combinations, these drugs display a broad spectrum of action against both Gram-positive and Gram-negative bacteria, effectively targeting the majority of intestinal pathogens that have been associated with UC and modulating bacterial enzymatic activities [122].

Antibiotics also possess potent anti-inflammatory and immunomodulatory properties [116,123]. Recent studies have shown that antibiotics can prevent tissue invasion and bacterial translocation, thus limiting systemic inflammation [116,124]. This approach has been used in the treatment of pediatric acute severe colitis [120,121].

Nevertheless, the therapeutic efficacy of antibiotics in UC is controversial due to its long-term impact on commensal microbes. Antibiotic treatments have been shown to significantly deplete microbial populations from colonic mucosae of IBD patients, and following cessation of therapy, commensal microbes undergo substantial structural and functional changes which may persist years after termination of the therapy [125]. Long-term exposure to antibiotic treatments impairs commensal bacterial diversity, leading to the abnormal proliferation of fungi, facilitating the growth of antibiotic-resistant species including methicillin-resistant *Staphylococcus aureus* (MRSA) and vancomycin-resistant *Enterococci* (VRE), and increasing susceptibility to secondary infections common in UC including *Clostridium difficile* colitis [117,125]. The effect of antibiotics on the structure and function of the commensal microbiome seems to be more significant when the therapy is administered during critical windows of early life development. Studies have also shown that children exposed to antibiotic therapies in early life are more susceptible to develop UC or CD in adulthood, implicating antibiotics as risk factors for autoimmune disease [126,127].

### 3.5. Fecal Microbiota Transplantation

Fecal microbiota transplantation (FMT) involves the transfer of prescreened intestinal bacteria from a healthy donor to an unwell recipient to restore the recipient microbiome to a healthier milieu and reduce symptoms associated with inflammation. The first use of FMT in human history dates to fourth century China for treatment of food poisoning [128]. Subsequent records show use during World War II for the treatment of bacterial dysentery [128]. Despite its long history, it was only in the past decade that FMT has gained recognition for its role in treating recurrent or refractory *Clostridioides difficile* infection (rCDI), with proven efficacy, safety, tolerability, and patient acceptance [128,129]. The effectiveness of FMT for rCDI is high, with several studies reporting >90% response after two administrations [130,131].

In response to its success for the treatment of rCDI, FMT has also received attention for its therapeutic potential in the treatment of UC. To date, one RCT in pediatric UC [132], and four RCTs in adult UC have assessed the role of FMT in treating chronic inflammation (Table 2) [133,134,135,136]. While methodologies across these studies are mixed, overall, FMT appears to show promise in inducing short-term remission [137]. In two qualitative studies exploring patient experiences with FMT in UC, patients reported positive experiences with treatment and an interest in receiving FMT in the future [138,139]. This high level of patient acceptance may further encourage research on the role of FMT in IBD therapy, leveraged by patient support groups and private foundations. FMT may also support cost-effectiveness as compared with conventional long-term UC therapies, as has been demonstrated in the treatment of rCDI [140].

Microbiome changes in adult and pediatric UC patients who received donor FMT suggests increased bacterial diversity, within 4–6 weeks post-transplant (Table 3) [132,133,135,141]. Paramsothy et al. and Costello et al. both reported an increase in donor-derived species from the *Prevotella* genus after 8 weeks [135,141] and *Anaerofilum pentosovorans* and *Bacteroides coprophilus* species were associated with disease improvement following FMT [141]. A decrease in *Bacteroides* genus, at 4 and 8 weeks, post FMT, as well as an increase in *Clostridium cluster XVIII* and *Ruminococcus spp* was associated with disease remission in recipients [145]. An increase in taxa typically found in the oral cavity, such as *Streptococcus spp* and *Fusobacterium spp,* was associated with lack of UC remission. Further, patients in remission after FMT had increased synthesis of SCFAs and secondary bile acids [145].

In the pediatric population, three of four studies reported some degree of clinical response post FMT [132,142,143]. Only one study reported adverse events such as worsening colitis requiring hospitalization for intravenous corticosteroid administration; this study by Pai et al. was also the only RCT to systematically assess the role of a FMT in pediatric UC patients using a placebo-controlled, blinded study design [132]. Among adult UC trials, 3 of 4 RCTs reported a statistically significant rate of achieving primary and secondary outcomes in the FMT group compared to control arms [133,135,141]. These four studies also employed the use of larger samples as compared with previous FMT studies and used pooled fecal matter from multiple donors to increase bacterial richness at baseline, during transplantation, and after treatment [132,133,135,141].

Various methods of FMT administration have been trialed. These include targeting upper GI routes via naso-gastric, naso-duodenal, and naso-jejunal tubes, as well as lower GI routes including colonoscopy infusions and enema-based therapies. More recently, oral capsules containing lyophilized or liquid FMT product have attracted interest for their ease of administration, convenient at-home use, and simple storage requirements [146,147]. Emerging evidence is showing impressive efficacy of capsule FMT for treatment of rCDI, with recent studies demonstrating equivalent clinical benefits and side effect profiles for capsule FMT as with traditional enema formulations [128,148,149]. To date, no study has been published assessing the efficacy of FMT capsules in the treatment of IBD. However, numerous studies using oral FMT capsular therapy are actively recruiting patients. The results of these studies will provide important information on the future of capsule FMT as a minimally invasive route of delivery, which will cater to patients’ growing interest for convenient at-home administration methods [138].

## 4. Microbial Influence on Progression to Colitis-Associated Cancer

### 4.1. Gut Dysbiosis and CAC

Longstanding UC correlates with an increased risk of developing CAC through cumulative inflammatory burden [150]. Complex interactions between various genetic and epigenetic factors, a Western diet high in refined sugars and animal fat, and low in dietary fiber and intestinal dysbiosis have been hypothesized to play key roles in tumorigenesis [4,151,152,153,154].

Gut dysbiosis may contribute to CAC through direct and indirect interactions with the host, such as bacterial metabolites and secreted molecules (e.g., genotoxins and virulence factors), attachment, invasion and translocation, and host defense modulation, leading to direct cell damage and chronic inflammation [155,156]. Among the different microbial-induced colon tumorigenesis theories, the alpha-bug hypothesis [157], driver-passenger hypothesis [158], and common ground hypothesis are most common [159].

In the alpha-bug hypothesis, a single pro-oncogenic microbe termed “alpha-bug” (particularly ﻿Enterotoxigenic *Bacteroides fragilis* (ETBF)) is thought to directly cause epithelial damage, modify colonic microbiota to further promote CAC development, and displace taxa that may protect against metaplasia. The driver-passenger model suggests that although a “driver bacteria” (with the same role as the alpha-bug) initially causes DNA damage, this results in microbial alterations that promote growth of opportunistic bacteria (i.e., bacterial passengers) which contribute to tumorigenesis. More recently, the common ground hypothesis has proposed that exogenous and endogenous factor (e.g., unhealthy diet, exogenous contaminants, and chronic inflammation) initially form a “leaky gut,” which results in transcellular hyperpermeability and bacterial internalization of pathobionts resulting in chronic inflammation and morphological changes in genetically predisposed individuals [157].

Bacteria such as ETBF, *Fusobacterium nucleatum*, *Escherichia coli*, and *Peptostreptococcus anaerobius* have been associated with colon cancer in human and animal models [109,160]. ETBF, through its zinc-metalloprotease toxin (*Bacteroides fragilis* toxin [BFT]), can trigger a carcinogenic inflammatory cascade by inducing E-cadherin cleavage, leading to increased intestinal permeability and Wnt/β-catenin and NF-κB signaling pathway activation, resulting in myeloid cell activation and increased levels of IL-17. This leads to a downstream series of immunological events that results in uncontrolled proliferation of colonic epithelial cells [161]. In addition, ETBF as well as polyketide synthase (*pks*)-positive *Escherichia coli*, have been associated with the creation of biofilms that coat adenomas, which may further promote tumorigenesis by altering the cancer metabolome (upregulation of *N*1,*N*12-diacetylspermine) and trigger IL-17-associated inflammation [162,163,164,165].

*Fusobacterium nucleatum*, a Gram-negative bacterium that resides in the oral cavity, has attracted interest over the past decade given its association with CAC. *Fusobacterium nucleatum* possesses several mechanisms that may contribute to CAC development [166]. *Fusobacterium nucleatum* adhesion protein A (*fadA*) facilitates attachment and invasion by binding to E-cadherin present in epithelial and malignant cells, resulting in expression of inflammatory molecules such as NF-κB, IL-6, IL-8, and IL-18, and TLR2/TLR4 activation [167,168]. *Fusobacterium nucleatum* has also been shown to accelerate DNA methylation in cancer-specific genes in patients with UC and appears to inhibit natural killer cell cytotoxicity via the Fap2 protein [169]. Colibactin-producing *Escherichia coli* (*Pks*^+^) has been shown to mediate cell damage through DNA alkylation and DNA double-strand breaks, contributing to tumorigenesis [110,170]. Colonic inflammation has been shown to further promote the genotoxic effects of *Pks*^+^ *Escherichia coli* [171]. *Peptostreptococcus anaerobius* has been shown to accelerate CAC in Apc^Min/+^ mice, and to attach to malignant cells via integrin α2/β1, a collagen receptor widely expressed on intestinal epithelial cells. This leads to downstream activation of the NF-κB pathway, a key regulator of intestinal inflammation and cancer development and progression [172].

A recent study in mouse CAC models found that α-diversity was decreased during the development of UC to CAC, and that the composition of the intestinal microbiome differed between three groups: control groups exhibited higher levels of Firmicutes, Verrucomicrobia, and Actinobacteria, and UC and CAC groups had higher levels of Proteobacteria, Firmicutes, and Verrucomicrobia [173]. Moreover, several metabolites were correlated with these microbial changes seen in the UC and CAC groups, specifically 12–hydroxy–8,10-octadecadienoic acid and linoleic acid positively correlated with *Enterobacteriaceae*, *Escherichia-Shigella*, and Proteobacteria. Thus, these metabolites could act as biomarkers for CAC.

Another animal study using azoxymethane/dextran sulfate sodium (AOM/DSS)-induced CAC murine models, showed that sucralose, a widely used caloric-free sweetener, led to an increase in the number and size of colonic tumors, inflammatory cytokines, and changes in the intestinal microbiota as compared with controls [174]. This highlights the importance of diet on the intestinal microbiota and CAC development.

In a recent study of patients with CAC, the CAC group was found to have decreased α-diversity, higher Proteobacteria, and decreased Firmicutes and Bacteroidetes as compared with healthy controls [175]. Significant differences were also found between the sporadic CRC group and the CAC group, with the latter having higher Proteobacteria, with *Bradyrhizobiaceae* and *Enterobacteriaceae* being the two overrepresented families. In addition, the levels of *Fusobacterium* were higher in the sporadic cancer group as compared with the CAC group. Further, there is evidence to suggest that the composition of the intestinal microbiota can change across different stages of CAC. In later-stage CAC, whereas *Akkermansia*, *Fusobacterium*, *Peptostreptococcus*, *Streptococcus*, and *Ruminococcus* were significantly higher, *Granulicatella* and *Lactobacillus* were significantly decreased as compared with non-CAC controls [176].

UC and CAC share similar microbial alterations that could potentially contribute to their shared pathogenesis. Whether these microbial alterations are the cause or consequence of chronic inflammation remains to be elucidated.

### 4.2. Intestinal Microbiota as an Emerging Target for the Treatment of Colitis-Associated Cancer

Given the potential role of the intestinal microbiome in the pathogenesis of CAC, gut bacteria-targeted therapies including probiotics, prebiotics, synbiotics, antibiotics, and FMT may hold promise [177,178,179]. This theory has strong biological plausibility. As we have discussed, mechanisms through which intestinal microbiota modulation occurs in CAC are similar to those seen in UC.

To the best of our knowledge, only two studies have been conducted on the use of FMT in murine models with CAC. Wang et al. used FMT to treat mice with AOM/DSS-induced CAC, which led to an increase in α-diversity as compared with the pre-FMT microbiota [173]. In addition, FMT led to an increase in colonic length, reduction in number of tumors and inflammation, as well as inhibition of proinflammatory molecules (IL-1β, IL-6, and TNF-α) and increase in anti-inflammatory cytokines (IL-10 and TGF-β). Furthermore, FMT-treated mice were found to have increased levels of CD3/CD4 in the lamina propria.

In another study of murine models with implanted colorectal adenoma cells and chemotherapy-induced mucosal injury, the authors found that FMT led to a reduction in diarrhea and intestinal mucositis, as well as suppression of IL-6 [180]. No significant differences were found in α-diversity between the groups.

Taken together, these findings suggest that FMT may be a promising therapy in modulating the intestinal microbiome of murine models with CAC. Larger studies are required to better understand the mechanisms and benefits of FMT in CAC.

## 5. Concluding Remarks

The intestinal microbiome exerts a major influence on the development and progression of UC and CAC. Our understanding of fungal and viral influences in the GI tract is steadily growing. With the support of culture-based sequencing, advanced metagenomics, and bioinformatics technologies, we are constructing a clearer picture of host–microbial dynamics. This provides more opportunities to understand disease pathogenesis at an individual level and may target treatments more effectively to individual patients’ UC and CAC biology.

While the cause of UC and CAC remains unclear, there is a clear role for the microbiome in regulating host inflammatory response and maintaining intestinal homeostasis. Our existing treatment paradigm of simply dampening immune activation through life-long, systemically acting immune suppression needs to keep pace with intestinal microbiome research. Multiple taxa have been implicated in triggering intestinal immune activation, and this is increasingly established through both structural and functional sequencing techniques. The metabolic contributions of key bacterial taxa play clear roles in epithelial cell function. The development of microbiota-based therapies will continue to have enormous potential. Exciting early data supports the role of FMT, prebiotics, probiotics, synbiotics, and select antibiotics in UC care.

Associations between microbial dysbiosis, chronic inflammation, autoimmunity, and tumorigenesis are well established. The future of GI pharmacotherapy will involve treatments that can halt this progression at its onset.

## Figures and Tables

**Table 1:** Intestinal microbiota alterations in ulcerative colitis and impacts on host immune, intestinal function.

Gut Microbiota Alterations in UC				Consequences for Mammalian Host Health	
Life Domain	Taxonomic Classification	Compositional Changes of Gut Microbiota	Functional Changes of Gut Microbiota	Impact on Host Immune Function	Impact on Host Intestinal Function
Bacteria	Phyla	16S ribosomal RNA gene sequencing ↓α- diversity in UC as compared with HC [70,78] ↑β-diversity in UC (UC bacteriome clusters differently form HC) [70,78] ↓relative abundance of Firmicutes and Bacteroidetes [20,70,71,74,81]	shotgun metagenomics sequencing ↑l-arginine biosynthesis (I, IV), biotin biosynthesis II, transfer RNA charging [78] Super pathway of polyamine biosynthesis in patients with risk factors for developing UC as compared with HC [78] ↑amino acid and protein metabolism (in UC as compared with HC): l-lysine fermentation to acetate and butanoate, creatinine degradation II, ketogenesis, protein *N*-glycosylation [77] ↑proteolytic and elastase activity in pre- and post-UC as compared with HC Correlated with the protease-producing bacterial species altered in UC- *Proteobacteria* and *Bacteroides*-↑elastase from *B. vulgatus*) [78] ↓glycerol and glycerophospholipids in UC as compared with HC Positive correlation between bacterial species and carbohydrate-degradation pathways [82]	*Ruminococcus, Eubacterium, Roseburia, and Akkermansia, Anaerostipes hadrus* ↓butyrate production = ↓Treg cells differentiation ↓maturation of Treg cells in the colonic epithelium increased levels of proinflammatory cytokines [71,72,78,79,83] *Enterobacteriaceae* ↑colonic epithelial cells invasion ↑levels of proinflammatory cytokine IL-8 and TNF-α [84] *Fusobacteria* ↑tumorigenesis in the colon [72] *Faecalibacterium prausnitzii* ↑production of IL-12, IFNγ and reduction of IL-10 levels in blood cells [85] *Adlercreutzia* ↓synthesis of isoflavones, phenolic compounds with antimicrobial and anti-inflammatory properties [78]	*Ruminococcus bromii, Eubacterium rectale, Roseburia, and Akkermansia* ↓butyrate production = impaired epithelial barrier function ↑epithelial permeability and commensals translocation [20,71,72] ↑colonic inflammation with crypt abscess [84] ↑of deciduous epithelial and/or blood cells in stools of patients with UC or CAC, gut barrier injury, impaired cell cycle [82]
		↑Proteobacteria [20,70,71,72,75]			
	Families	↓*Clostridiaceae* [71,72] ↑*Enterobacteriaceae* [86]			
	Genera	↓*Clostridium clusters IV, XIVa* [72] ↓*Ruminococcus, Eubacterium, Roseburia, Akkermansia* [71,78] ↓*Adlercreutzia, Bilophila, Bifidobacterium* [78] ↓*Bacteroides, Lachnospira, Phascolarctobacterium, Coprococcus, Odoribacter, Butyricimonas* [75,86]			
		↑*Escherichia-Shigella, Fusobacterium,* *Campylobacter, Helicobacter* [71,75,78] ↑*Actinobacillus* [78] ↑*Streptococcus, Anaerostipes Enterococcus, Actyinomyces, Lactobacillus, Acetobacter, Rothia, Pseudomonas, Collinsella* [75]			
	Species	*↓Faecalibacterium prausnitzii* [72,83,87] *↓Anaerostipes hadrus* [79] ↑*Flavonifractor plautii, Coprococcus catus, Parabacteroides merdae* [78]			
Fungi	Phyla	Stool ITS2 gene sequencing *↓*α-diversity in UC (not in CD) [70] ↑β-diversity between UC in flare as compared with UC in remission and to HC [70] ↑ ratio of Basidiomycota/Ascomycota in UC in flare as compared with UC in remission and to HC [70] ↑correlation between fungi and bacteria in UC as compared with CD and HC [70] Colonic mucosa: ↓fungi load in UC as compared with HC No significant changes in α-diversity UC mycobiota clusters differently from HC No changes in the ratio of Basidiomycota/Ascomycota [88]	N/A	*Saccharomyces cerevisiae* and *Candida Albicans =* ↑IL-6 production [70] *↓Saccharomyces cerevisiae =* ↓IL-10 production (anti-inflammatory cytokine) [70] *Aspergillus* ↑aflatoxin production, a carcinogenic mycotoxin [88] Positive correlation between *Wickerhamomyces* and *Penicillium* with the expression of TNF-α and IL-17A, respectively (in colonic mucosa) [88] Negative correlation between *Sporobolomyces* and IL-6 and between *Trametes* and IL-1β (in colonic mucosa) [88]	*Aspergillus* Potential for aspergillosis, with consequent abdominal pain and GI bleeding [88]
	Genera	*↓Saccharomyces* in UC fecal samples [70] ↑*Aspergillus* in UC mucosa specimen [88]			
	Species	*↓Saccharomyces cerevisiae* in UC fecal samples [70] ↑*Candida albicans* in UC fecal samples [70] Trend toward an increase in mucosal specimen [89]			
Virus	Orders	Metagenomics sequencing of viral-like particles *↓*α-diversity (virome species richness and evenness) in UC mucosal samples [90] ↑abundance Caudovirales bacteriophages in UC mucosal samples [90] ↑β-diversity; UC mucosal virome clusters differently from HC [90] ↑virome dissimilarity between UC subjects (not observed in HC subjects) [90]	↓integral component of membrane, DNA binding, ATP-binding cassette (ABC) transporter and integrase core domain in UC as compared with HC [90] ↑Pathways related to the phage lysis of bacteria: DNA template negative regulation of transcription, beta-lactamase, glutamine amidotransferase, glycosal hydrolases, type II/IV secretion system and multicopper oxidase in UC as compared with HC [90]	↑bacteriophage = ↑bacterial lysis, PAMPs production, TLRs overstimulation, ↑intestinal inflammation [90] ↑transfer of bacterial genetic material (i.e., antibiotic resistance genes) [90] ↑phages can stimulate IFN-γ via the nucleotide-sensing receptor TLR9 [91]	↑bacteriophages = ↑bacterial lysis, ↑intestinal inflammation, potential implication in abdominal pain, diarrhea [90,91]
	Families	*↓Anelloviridae* (eukaryotic virus) [90] ↑*Microviridae* (single-stranded DNA phage), *Myoviridae, Podoviridae* (double-stranded DNA phages) [90] *Pneumoviridae* (eukaryotic virus) [90]			
	Genera	*↓Coccolithovirus* , *Minivirus* *Orthopoxvirus* (vertebrate-infecting virus) (all eukaryotic viruses) [90] ↑*Phix174microvirus*, *P1virus, Lambdavirus*, *T4virus, P22virus* (all *Caudovirales* bacteriophages) *Orthopneumovirus* [90]			
	Species	*↓*α-diversity of *Caudovirales* species in UC mucosal samples [90] ↑*Escherichia* and *Enterobacteria* bacteriophages [90] *Lactobacillus, Escherichia,* and *Bacteroides* bacteriophages [91]			

UC, ulcerative colitis; HC, healthy controls; CD, Crohn’s disease; IL, interleukin; CAC, colitis-associated cancer; IFN, interferon; TNF, tumor necrosis factor-α; Treg, regulatory T-cell.

**Table 2:** Summary of methods, outcomes, and results in adult, pediatric fecal microbiota transplant studies.

Primary Author (Year)	Country	Study Type	Population	Study Characteristics: *n*, Sex, Years (Range)	Donor Characteristics; FMT Preparation	FMT Route of Administration	Methods: FMT, Outcomes	Pre-Administration Preparation	Outcomes: Primary, Secondary	Key Findings, Adverse Events	Strengths, Limitations
Adult Studies											
Costello et al. (2019) [141]	Australia	Multicenter, double-blind, placebo-controlled RCT	Adult UC patients (Mayo score = 3–10, endoscopic subscore ≥2)	Sample: *n* = 73 (38 dFMT; 35 aFMT) Sex: 40 males, 33 females Age: Treatment group = 38.5 (28–52); Control group = 35 (25–46)	Donors: 19 anonymous donors (age 18–65),pooled fecal matter from 3–4 donors Preparation: Stool frozen at −80 °C, thawed before administration	Colonoscopy	Administration: 200 mL fecal suspension of dFMT or 200 mL aFMT delivered to right colon, followed by 100 mL of dFMT/aFMT enema x 7 days Outcome Data: Recipient stool samples collected at baseline, 4, 8, 52 weeks Sent for microbiome, metabolome, fecal calprotectin assessment Mucosal biopsies via colonoscopy at Weeks 0 and 8At 8 weeks, open-label dFMT offered to control participants and followed × 12 months	3 L polyethylene glycol evening before administration Loperamide 2 mg orally before colonoscopy	Primary: Steroid-free remission Mayo score ≤2, with endoscopic Mayo subscore ≤ 1 at Week 8 Secondary: Clinical response (≥ 3-point reduction in Mayo score at Weeks 8 and 12) Clinical remission (SCCAI ≤2 at Week 8 and 12 months) Participant perception, acceptance of FMT via survey at baseline and 12 months Adverse events via survey at 8 and 12 months	Primary: 12/38 (32%) dFMT group vs. 3/35 (9%) aFMT group5/12 Participants (42%) who achieved primary endpoint at 8 weeks from dFMT group maintained remission at 12 months Secondary: 21/38 (55%) dFMT group vs. 8/35 (23%) aFMT group achieved clinical response 18/38 (47%) dFMT group had clinical remission vs. 6/35 (17%) aFMT group4/38 (11%) dFMT group had endoscopic remission vs. 0/35 (0%) aFMT group72/73 (99%) received dFMT at 12 months Adverse Events: 3 SAEs dFMT group: 1 worsening colitis, 1 *C. Difficile* colitis requiring colectomy, 1 pneumonia 2 SAEs aFMT group: 2 worsening colitis	Strengths: Anaerobic stool processing of dFMT/aFMT, stool collections preserve obligate anaerobes Pooled fecal donors increase diversity of donor taxa Limitations: No prior antibiotic washout period 12-Month outcome data limited by open-label crossover study design, observational only Significant loss of follow-up at 12 months
Moayyedi et al. (2015) [133]	Canada	Single center, double-blind, placebo-controlled RCT	Adult UC patients (Mayo score ≥4, endoscopic subscore ≥1)	Sample: *n* = 75 (38 dFMT; 37 placebo) Sex: 44 males, 31 females Age: Treatment group = 42.2; Control group = 35.8	Donors: 5 anonymous donors, 1 family member (age 18–60), fecal matter from a single donor Preparation: Stool administered within 5 h of collection or frozen at −20 °C, thawed before administration	Retention Enema	Administration: 50 mL dFMT or 50 mL water administered × 6 weeks Outcome Data: Mayo clinic score, IBDQ, EQ-5D, flexible sigmoidoscopy at week 7 Rectal, sigmoid, descending colon biopsies via colonoscopy at baseline, Week 7 Stool sample collected weekly prior to enema administration Stools sent for 16s rRNA sequencing	No pre-FMT prep was done	Primary: Remission of UC (Mayo score ≤2) Complete healing of mucosa seen on flexible sigmoidoscopy at 7 weeks (endoscopic Mayo score of 0) Secondary: Improvement in UC Symptoms (≥3 improvement in full Mayo score) Change in Mayo, IBDQ, EQ-50 scores Adverse events	Primary: 9/38 (24%) dFMT group vs. 2/37 (5%) in the placebo group Secondary: Improvement in symptoms and quality of life scores were not statistically significant Immunosuppressant therapy had greater benefit from dFMT than those not on immunosuppressive therapy (5/11 (46%) vs. 4/27 (15%)) Participants with recent diagnosis of UC (≤ 1 yr) were more likely to respond to dFMT (3/4 (75%)) than those with longer disease duration (>1 year) (6/34 (18%)) Frozen stool had greater efficacy than fresh stool Adverse Events: 3 SAEs dFMT group: 2 colonic inflammation and rectal abscess formation, 1 worsening abdominal discomfort with C. Difficile diagnosed after study exit 2 SAEs placebo group: 1 worsening colitis with admission and emergency colectomy, 1 colonic inflammation and rectal abscess formation	Strengths: Large sample size as compared with previous studies Limitations: No bowel preparation Participants with extensive colitis could have active disease beyond visualization of sigmoidoscopy
Paramsothy et al. (2017) [135]	Australia	Multicenter, double-blind, placebo-controlled RCT	Adult UC patients (Mayo score = 4–10, endoscopic subscore ≥1, physician’s global assessment subscore ≤2)	Sample: *n* = 81 (41 dFMT; 40 placebo) Sex: 47 males, 34 females Age: Treatment group = 35.6 (27.8–48.9); Control group = 35.4 (27.7–45.6)	Donors: 14 anonymous donors, pooled fecal matter from 3–7 donors Preparation: Stool frozen at −80 °C, dispensed for home freezer storage at −20 °C	Colonoscopy + Enema	Administration: 150 mL dFMT or 150 mL isotonic saline 5 days per week × 8 weeks Outcome Data: Stooling frequency, haematochezia, miscellaneous gastrointestinal symptoms, medication changes At 8 weeks, open-label dFMT was offered to participants in the placebo group	Not specified	Primary Outcomes: Steroid-free clinical remission with endoscopic remission or response at Week 8 Mayo score ≤2, all subscores ≤1, ≥1 point reduction in endoscopy subscore Secondary Outcomes: Steroid-free clinical remission (combined Mayo subscore of ≤1 for rectal bleeding + stool frequency) Steroid-free clinical response (decrease of ≥3 on Mayo score OR ≥ 50% reduction from baseline combined with rectal bleeding + stool frequency Mayo subscore OR both) Steroid-free endoscopic subscore of ≤1 with a reduction ≥1 point from baseline Steroid-free endoscopic remission (Mayo endoscopy subscore of 0) Quality of life (IBDQ) Adverse events	Primary Outcomes: 11/41 (27%) dFMT group vs. 3/40 (8%) in the placebo group Endoscopic remission did not differ between study groups (steroid-free Mayo endoscopic subscore of 0) 3x greater endoscopic response in dFMT group (32% (13/41) vs. 10% (4/40)) Secondary Outcomes: 18/41 (44%) steroid-free clinical remission in the dFMT group vs. 8/40 (20%) in the placebo group 22/41 (54%) steroid-free clinical response in the FMT group vs. 9/40 (23%) in the placebo group 13/41 (32%) steroid-free endoscopic response in the FMT group vs. 4/40 (10%) in the placebo group, but no difference in endoscopic remission Adverse Events: 2 SAEs dFMT group: 1 clinical and endoscopic deterioration with colectomy, 1 unwell and admitted for intravenous corticosteroid therapy 1 SAE placebo group: hospitalisation, reason not stated	Strengths: Large sample size Intensive dosing schedule (40 infusions over 8 weeks) Multidonor dFMT had greater microbial diversity than single donor dFMT Limitations: Mandatory steroid-wean clinically demanding, resulted in many withdrawals from study Enema preparations challenging and inconvenient for self-administration Use of multidonor batches prevented analysis of dononspecific factors associated with therapeutic outcomes
Rossen et al. (2015) [134]	Netherlands	Single center, double-blind, placebo-controlled RCT	Adult UC patients (Lennard-Jones Criteria, patient reported SCCAI ≥4 and ≤11)	Sample: *n* = 48 (23 dFMT; 25 aFMT) Sex: 22 males, 26 females Age: Treatment group = 40 (33–56); Control group = 41 (30–48)	Donors: 15 anonymous donors, 1 family member, fecal matter from a single donor Preparation: Stool administered within 6 h of preparation	Nasoduodenal Tube	Administration: 500 mL dFMT or aFMT administered at baseline, 3 weeks Outcome Data: Clinical, colonoscopic follow-up at 6 weeks and 12 weeksFecal samples at baseline and prior to each dFMT/aFMT treatment	2 L macrogol solution (MoviPrep^®^) 2 L clear fluids evening before administration	Primary Outcomes: Clinical remission (SCCAI ≤2 and ≥1 point decrease in Mayo endoscopic score) at Week 12 Secondary Outcomes: Clinical response (reduction of ≥1.5 points on SCCAI) Clinical remission (SCCAI ≤2) Endoscopic response Change in median IBDQ score from baseline to Week 6 Microbiota composition by phylogenic microarray in fecal samples	Primary Outcomes: No statistically significant difference in clinical and endoscopic remission between study groups (trial was stopped early due to interim results suggesting the study would not lead to a statistically significant outcome) Secondary Outcomes: At 12 weeks, 11/23 (47.8%) dFMT participants and 13/25 (52%) aFMT participants had a clinical response 3 SAEs reported but treatment allocation group not specified for all: 1 was admitted to hospital and diagnosed with small bowel Crohn’s disease, 1 developed cytomegalovirus infection (aFMT group), 1 was admitted for abdominal pain	Limitations: Small sample size Low FMT dosing regimen (2 FMTs, 3 weeks apart)
**Pediatric Studies**											
Pai et al. (2021) [132]	Canada	Multicentre, single-blind, placebo-controlled RCT	Pediatric UC patients with mild-severe disease (PUCAI ≥ 15 and elevated fecal calprotectin, or CRP)	Sample: *n* = 25 (13 FMT; 12 controls) Sex: 13 males, 12 females Age: 12.2 (4–17)	Donors: FMT products obtained from Rebiotix, Inc. Stool pooled from anonymous donors Administration: Stool frozen at −80 °C, then refrigerated (4 °C) for up to 3 days until administration	Enema	Administration: 150 mL FMT, or 150 mL normal saline 2×/week × 6 weeks Outcome Data: BloodworkPUCAI Fecal calprotectin, microbiome analyses (Above) measured 2×/week × 6 weeks, then, weeks 12/18/24/30	No pre-FMT prep was done	Primary: Recruitment rate Secondary: Clinical remission = decrease in PUCAI to <10 Clinical response = decrease in PUCAI by ≥15 Biological improvement (decreased CRP, fecal calprotectin)Composite clinical response = reduction from baseline in FC, CRP, PUCAI score Changes in microbiota	Outcomes: Primary feasibility outcome (achieving recruitment target) not reached11/12 (92%) dFMT group had improvement in PUCAI, CRP, fecal calprotectin from baseline vs. control group (6/12 (50%)) at Week 69/12 (75%) maintained clinical response at 12 months Adverse Events: 5 SAEs dFMT group: 3 worsening colitis requiring hospitalization for intravenous corticosteroids, 2 *C. Difficile* diagnosed after study exit (not detected in dFMT sample) 1 SAE control group: 1 worsening colitis requiring hospitalization for intravenous corticosteroids	Strengths: First multi-center, placebo-controlled blinded RCT in pediatric UC Open-label study design offered to control group at completion Largest sample size as compared with previous pediatric studies Limitations: Lack of endoscopic outcomes Lack of investigator blinding
Kellermayer et al. (2015) [142]	USA	Prospective, open-label case series	Pediatric UC patients (mild-severe)	Sample: *n* = 3 Sex: 2 males, 1 female Age: 15 (14–16)	Donors: Stool obtained from a single anonymous donor Administration: Stool frozen until administration	Colonoscopy + Enema	Administration: Tapering course (22–30 treatments) FMT over 6–12 weeks Outcome Data: Mucosal disease activity (colonoscopy), PUCAI, Mayo score, fecal microbiome at baseline, 2 weeks after FMT	Not specified	Mucosal disease activity before, 2 weeks after FMT treatments PUCAI Changes in microbiota	Outcomes: All participants in endoscopic and clinical remission 2 weeks after the last FMT Adverse Events: None	Limitations: Small sample size Lack of randomization
Kunde et al. (2013) [143]	USA	Prospective, open-label case series	Pediatric UC patients (mild-moderate; PUCAI 15–65)	Sample:*n* = 10 Sex: 6 males, 4 females Age: 15.2 (7–20)	Donors: Stool obtained from family members or close friends Administration: Stool administered within 6 h of preparation	Retention Enema	Administration: FMT (administered over 1 h) daily × 5 days (60 mL administered every 15 min) Outcome Data: PUCAI, patient acceptance/tolerability at baseline, weekly × 4 weeks after FMT	No pre-FMT prep was done	Clinical response = decrease in PUCAI by >15 after FMTClinical remission = decrease in PUCAI to <10Clinical endpoint: clinical response at 1 month post FMTAdverse events	Outcomes: 7/9 (78%) showed clinical response within 1 week −6/9 (67%) maintained clinical response at 1 week 3/9 (33%) achieved clinical remission at 1 week and remained remission at 4 weeks Adverse Events: None	Limitations: Small sample size Children with mild-to-moderate disease
Suskind et al. (2015) [144]	USA	Prospective, open-label case series	Pediatric UC patients (mild-moderate)	Sample:*n* = 4 Sex: 4 males Age: 14.5 (13–16)	Donors: Further details not available Administration: Further details not available	Nasogastric Tube	Administration: 30 mg of donor stool mixed with 100 mL normal saline, infused over 3 min, ollowed by saline flush over 1 min Outcome Data: PUCAI, CRP, fecal calprotectin at baseline (Above) measured at week 2/6/12	Rifaximin (200 mg three times daily × 3 days) 1 capful of MiraLAX^®^ in water 3 times daily × 2 days) Omeprazole (1 mg/kg orally) on the day before, morning of procedure	Clinical remission = decrease in PUCAI to <10adverse events	Outcomes: None of the participants clinically improved No significant change in PUCAI scores, CRP, or stool calprotectin at 2 weeksNo significant changes to albumin or haematocrit Adverse Events: None	Limitations: Small sample sizeFMT via nasogastric tube may have altered microbiota diversity

Litre; FMT, fecal microbiota transplant; dFMT, donor FMT; aFMT, autologous FMT; SCCAI, Simple Clinical Colitis Activity Index; SAE, serious adverse events; IBDQ, Inflammatory Bowel Disease Questionnaire; EQ-5D, EuroQol-5D; PUCAI, Pediatric Ulcerative Colitis Activity Index; RCT, randomized controlled trial.

**Table 3:** Summary of microbial changes in adult, pediatric fecal microbiota transplant studies.

Primary Author (Year)	Microbial Changes			
Adult Studies				
Costello et al. (2019) [141]	Sequencing technique: 16S ribosomal RNA sequencing (V4 region of 16S ribosomal RNA gene) Effect of FMT on bacterial diversity: Baseline: Bacterial diversity was highest in blended donor stool, then individual donor stool and stool from UC patients Weeks 4 and 8: bacterial diversity of stool increased in dFMT vs. aFMT group, but no significant difference was reported Effect of FMT on bacterial taxa abundance: Increased relative abundance of bacterial taxa following dFMT (as compared with aFMT) up to 8 weeks			
	**Phyla**	**Families**	**Species**	
	↑Firmicutes	*Peptococcaceae* *Erysipelotrichaceae* *Acidaminococcaceae* *Ruminococcaceae*	*Peptococcaceae* *Faecalicoccus pleomorphus* *Acidaminococcus intestini* *Clostridium methylpentosum*	
	↑Bacteroidetes	*Prevotellaceae* *Rikenellaceae* *Porphyromonadaceae*	*Prevotella copri* *Alistipes indistinctus* *Odoribacter splanchnicus strain*	
	↑Actinobacteria	*Coriobacteriaceae*	*Olsenella sp.* *Senegalimassilia anaerobia* *Slackia isoflavaniconvertens*	
	↑Euryarchaeota	*Methanobacteriaceae*	*Methanobrevibacter smithii*	
	Decrease in relative abundance of bacterial taxa following dFMT (as compared with aFMT) up to 8 weeks:			
	**Phyla**	**Families**	**Species**	
	↓Firmicutes	*Lachnospiraceae*	*Anaerostipescaccae* *Clostridium aldenense*	
	↓Actinobacteria	*Coriobacteriaceae*	*Gordonibacter pamelaeae*	
	Strong association between *Anaerofilum pentosovorans* (phylum Firmicutes) and *Bacteroides coprophilus* (phylum Bacteroidetes) with disease improvement after dFMT			
Moayyedi et al. (2015) [133]	Sequencing technique: 16S ribosomal RNA sequencing (V3 region of 16S ribosomal RNA gene) Effect of FMT on bacterial diversity: Greater bacterial diversity in the dFMT as compared with a placebo group at Week 6 vs. baseline (*p* = 0.02, Mann–Whitney U test) dFMT group had more similarities in taxonomic profile to their donor than placebo group Effect of FMT on bacterial taxa abundance Two major donors (A and B) showed different bacterial composition Donor B: *↑Lachnospiraceae* family and *↑Ruminococcus genera*; Donor A: *↑Escherichia* and *Streptococcus genera* Donor B was associated with successful FMT; the microbial profile of dFMT responders from Donor B was similar to that of Donor B, but did not match among non-responders			
Paramsothy et al. (2017, 2019) [135,145]	Sequencing technique: 16S ribosomal RNA sequencing (V4 region of 16S ribosomal RNA gene) (Paramsothy et al. 2017); shotgun metagenomics (Paramsothy et al. 2019) Effect of FMT on bacterial diversity: Increased phylogenetic diversity after 4 and 8 weeks of FMT as compared with the baseline Increased α-diversity after dFMT (as compared with a placebo), in both stool and mucosal biopsies Increased β-diversity after dFMT (as compared with the baseline and placebo), in both stool and mucosal biopsies dFMT patients who achieved primary outcome have higher fecal species richness than at baseline, during FMT therapy, and after therapy as compared with those who did not achieve primary outcome Effect of FMT on bacterial taxa abundance: Increased donor-derived *Prevotella* genus and a decrease in baseline patient-derived *Bacteroides* genus after 4 and 8 weeks of FMT Bacterial taxa associated with remission after double-blind FMT: *Barnesiella spp, Parabacteroides spp*, *Clostridium cluster IV*, and *Ruminococcus spp* Bacterial taxa associated with remission after open-label FMT: *Blautia spp, Dorea spp, Ruminococcus 2*, and *Clostridium cluster XVIII* *Fusobacterium spp* and *Sutterella spp* (phyla *Fusobacteria* and *Proteobacteria*) were associated consistently with no remission Patients in remission after FMT: ↑*Eubacterium hallii, Roseburia inulivorans, Ruminococcus bromii* (phylum Firmicutes), *Eggerthella* species (phylum Actinobacteria), ↑*Oscillibacter, Clostridium XVIII*, *Roseburia* (phylum Firmicutes) in stool and mucosa biopsies associated with primary outcomes Increased short-chain fatty acid biosynthesis and secondary bile acids Patients not in remission after FMT: ↑ *Fusobacterium gonidia-formans, Sutterella wadsworthensis, Haemophilus, Escherichia species, Prevotella*, *Bilophila* (phylum Proteobacteria) Pathways associated with a negative therapeutic outcome including heme, lipopolysaccharide, and peptidoglycan biosynthesis contribute to bacterial virulence and increased inflammation *Streptococcus* species (phylum Firmicutes), commonly implicated with the oral cavity, associated with lack of remission Other oral bacterial taxa such as *Dialister*, *Veillonella*, and *Parvimonas* (phylum Firmicutes) were associated with a negative patient outcome			
Rossen et al. (2015) [134]	Sequencing technique: 16S ribosomal RNA sequencing Effect of FMT on bacterial diversity: Baseline: stool bacterial composition of healthy donors is more stable than UC patients; no difference in α-diversity between healthy donors and UC patients 12 Weeks: increased bacterial richness and evenness in both dFMT and aFMT groups (responders); bacterial composition of dFMT responders more similar to healthy donors; aFMT composition is different from healthy donors and dFMT Effect of FMT on bacterial taxa abundance:			
	UC patients before dFMT **Phylum**		**Genus**	
	Firmicutes		↓*Clostridium* cluster *IV*, *XIVa*, *XVIII* ↑*Clostridium* clusters *IX*, and *XI*; *Bacillus*	
	↑Bacteroidetes ↑Proteobacteria			
	UC patients after dFMT:			
	**Phylum**		**Genus**	
	Firmicutes		↑*Clostridium* cluster *IV, XIVa, XVIII*	
	↓Bacteroidetes			
	UC patients after aFMT:			
	**Phylum**		**Genus**	
	Firmicutes ↑Bacteroidetes ↑Proteobacteria		↑*Bacillus*	
**Pediatric Studies**				
Pai et al. (2021) [132]	Sequencing technique: 16S ribosomal RNA sequencing (V3 region of 16S ribosomal RNA gene) Effect of FMT on bacterial diversity: Increased β-diversity observed in dFMT after 6 weeks from baseline as compared with a placebo group Effect of FMT on bacterial taxa abundance:			
	Bacterial changes positively correlated with an increase in CRP, Fcal → improvement of colitis symptoms **Phylum**	**Order**	**Family**	**Genus**
	↑Firmicutes	↑Clostridiales	*↑Ruminococcaseae* *Lachnospiraceae* *Peptostreptococcaceae* *Erysipelotrichaceae*	*↑* *Ruminococcaceae* *Coprococcus* *Romboutsia* *Erysipelotrichaceae*
	↑Bacteroidetes	↑Bacteroidales	*↑* *Rikenellaceae*	*↑* *Alistipes*
Kellermayer et al. (2015) [142]	Sequencing technique: 16S ribosomal RNA sequencing (V3V5 regions of 16S ribosomal RNA gene) Effect of FMT on bacterial diversity: Increased bacterial richness and diversity in stool after FMT Effect of FMT on bacterial taxa abundance:			
	**Phylum**	**Family**	**Genus**	
	Firmicutes	↑*Lachnospiraceae*	↑*Coprococcus*	
	Inversely correlated with UC disease activity Beneficial effect of *Coprococcus* (butyrate-producing bacteria) to the colonic epithelium of UC patients			
Kunde et al. (2013) [143]	Not applicable			
Suskind et al. (2015) [144]	Not applicable			

RNA, ribonucleic acid; FMT, fecal microbiota transplant; dFMT, donor FMT; aFMT, autologous FMT; CRP, C-reactive protein; Fcal, fecal calprotectin; UC, ulcerative colitis.

## Data Availability

Not applicable.
